# Vδ2 T-cells response in people with Mpox infection: a three-month longitudinal assessment

**DOI:** 10.1080/22221751.2025.2455585

**Published:** 2025-01-16

**Authors:** Eleonora Cimini, Eleonora Tartaglia, Francesco Messina, Andrea Coppola, Valentina Mazzotta, Massimo Tempestilli, Giulia Matusali, Stefania Notari, Annalisa Mondi, Gianluca Prota, Alessandra Oliva, Carla Fontana, Enrico Girardi, Fabrizio Maggi, Andrea Antinori

**Affiliations:** a Laboratory of Cellular Immunology and Pharmacology, National Institute for Infectious Diseases “Lazzaro Spallanzani” IRCCS, Rome, Italy; b Laboratory of Virology and Biosafety Laboratories, National Institute for Infectious Diseases “Lazzaro Spallanzani” IRCCS, Rome, Italy; c Microbiology Laboratory and Biobank, National Institute for Infectious Diseases, Lazzaro Spallanzani IRCCS, Rome, Italy; d HIV/AIDS Unit, National Institute for Infectious Diseases “Lazzaro Spallanzani” IRCCS, Rome, Italy; e Scientific Direction, National Institute for Infectious Diseases, Lazzaro Spallanzani IRCCS, Rome, Italy

**Keywords:** Innate immunity, Vδ2 T-cells, emerging viruses, Mpox, exhaustion

## Abstract

The first evidence that Orthopoxvirus induced the expansion *in vivo* and the recall of effector innate Vδ2 T-cells was described in a macaque model. Although, an engagement of αβ T-cells specific response in patients infected with human monkeypox (Mpox) was demonstrated, little is known about the role of γδ T-cells during Mpox infection. IFN-γ-producing γδ T-cells in the resistance to poxviruses may a key role in inducing a protective type 1 memory immunity. We analyzed the kinetics of Vδ2 T-cell response from the acute phase up to three months after Mpox infection. Fourteen MSM subjects (5 PWH, 35.7%) were enrolled in a longitudinal study from May to July 2022. Blood samples were collected in the early phase of infection (T1, T2) and at 3 months (T3M) post-symptom onset. Vδ2 T-cell profiles (CD45RA/CCR7), activation/exhaustion markers (CD38/HLA-DR/CD57/PD-1/TIM-3), cytokine production (IFN-γ/TNF-α) and CD107a expression were assessed by multiparametric flow cytometry. Ten healthy donors (HD) were used as a control group. At T1, Vδ2 T-cell frequency of patients decreased, and effector memory Vδ2 T-cells increased with respect to HD. Activation/exhaustion markers were higher than HD. Vδ2 functionality decreased at T1 related to HD, and it was associated with CD38 and HLA-DR higher expression as well as TIM-3. Vδ2 T-cells restored their profile at T3M. The presence of effector/activated Vδ2 T-cells in the early stages of Mpox infection and their capability to activate quickly, producing pro-inflammatory cytokines, may be useful to enhance the early adaptive response to human Mpox, maintaining a protective memory/effector T-cell response.

## Introduction

Mpox (formerly known as monkeypox) is a viral zoonosis historically spreading in Central and West Africa. The etiological agent of Mpox is the monkeypox virus (MPXV), a DNA virus of the *Poxviridae* family (genus Orthopoxvirus) [1]. The infection follows two transmission lanes, e.g. [1] animal to human (via direct contact or through the ingestion of the meat of an infected animal) and [2,3] human to human (via direct contact with skin lesions, body fluids, and infected people’s contaminated objects).

In May 2022, the World Health Organization declared the human Mpox outbreak in non-endemic countries a public health emergency. The 2022 outbreak was primarily driven by the transmission of the virus via intimate contact in men sexually active with other men, with a higher HIV frequency among them. As of April 2024, a total of 27,180 cases of Mpox have been identified in 46 countries and areas throughout the European Region [4]. Most cases were males (98%) and men sexually active with other men (96%). Among cases with known HIV status, 38% were persons living with HIV (PWH) [4], highlighting the need for vaccination strategies for high-risk populations [5,6]. Clinical features were well characterized, and most cases had a cutaneous rash (95%) and systemic symptoms (67%), such as fever, fatigue, muscle pain, chills, or headache. Hospitalized persons were about 7% of all notified cases in Europe; eight cases were admitted to the intensive care unit, and 10 deaths of cases of Mpox have been currently documented [4].

This epidemiological change of Mpox raises concerns for an increasing spread of the disease in non-endemic countries and urges a plan for controlling viral circulation, enhancing vaccine strategies. Understanding the involvement of the immune response is important in defining the host’s response to infection and thus the window of action for possible vaccine strategies.

The gamma-delta (γδ) cells are unique T-cells that belong to the innate immune system. In both humans and mice, γδ T-cells are a minor part (1–5%) of the T-cells circulating in peripheral blood and in secondary lymphoid organs. Human γδ T-cells can be divided into four major populations (δ1, δ2, δ3, and δ5), based on the T-cell receptor δ (TCRδ) chain expression [7]. The Vδ9 T-cells co-expressing the Vδ2 chain [8,9] are the most common subpopulation of γδ T-cells in the peripheral blood and secondary lymphoid organs of adult individuals. Vγ9Vδ2 T-cells are primarily localized in peripheral blood, and, following pathogen injury, they can be recruited to inflamed tissues and participate in pathogen and tumour clearance [10]. Vγ9Vδ2 T-cells can be activated by small phosphorylated compounds called phosphoantigens (P-Ags) [11,12], described in the mevalonate isoprenoid pathway in mammalian cells, and the most representative is isopentenyl-pyrophosphate (IPP) [12].

During an infection, γδ T-cells may be activated, resulting in a strong and rapid effector response mediated by cytotoxic granules and inhibitory or lytic molecules [13]. Moreover, γδ T-cells have an indirect role in the elimination of microorganisms via the production of pro-inflammatory cytokines and the induction of antimicrobial functions in immune and epithelial cells [13,14]. An expansion of γδ T-cells in peripheral-blood mononuclear cell samples was reported in non-vaccinated volunteers stimulated with Vaccinia virus. These findings demonstrate a potent soluble Vδ2-antiviral activity *in vitro* against the Vaccinia virus [15] and suggest the involvement of γδ T-cells in the Orthopoxvirus-induced immune response. In addition, the first evidence that Orthopoxvirus elicited the *in vivo* expansion and the recall of effector Vδ2 T-cells was described in a macaque model [16]. However, *in vivo* data on circulating γδ T-cell response in human Mpox infection are lacking. In filling this knowledge gap, it is important to assess if enhancing the γδ T-cell response can represent an important issue for understanding pathogenesis/protection mechanisms during Mpox infection.

Motivated by this notion, this study assessed the three-month post-acute infection kinetics of the Vδ2 T-cell innate response in people with Mpox, testing the hypothesis that Vδ2 T-cells may play an important role in host protection during Mpox infection.

## Material and methods

### Study design and cohort

This study was performed at the National Institute for Infectious Diseases Lazzaro Spallanzani, Rome, Italy. The local ethical committee approved the study (Mpox-Cohort, approval number 40z_2022 plus amendment n. 1, approval number 6z_2023), and all participants signed an informed consent. Fourteen people with a laboratory-confirmed diagnosis of Mpox infection were consecutively enrolled. Five were people living with HIV (PWH). Subjects were required to undergo a blood draw at the time of the acute infection and at the following post-symptom onset time points: day 3–4 (T1), day 15–20 (T2), and month three (T3M). Ten age-matched healthy donors (HD) were enrolled as the control group. Table 1 describes the clinical and biological features of the study cohort.

**Table 1.** Clinical and biological features of the study cohort.

	*Patients*
Age, median (range, years)	41 (32–51)
Male, n (%)	14 (100%)
People living with HIV, *n* (%)	5 (37.5%)
Sexual transmission, *n* (%)	14 (100%)
MSM, *n* (%)	13 (88%)
PWH under antiretroviral therapy, *n* (%)	5 (100%)
Systemic Symptoms, *n* (%)	14 (100%)
Mpox treatment, *n* (%)	1 (7.1%)
Smallpox Vaccination, *n* (%)	1 (7.1%)
CD4 count median (cells/mm^3^)	670 (413–972)
HIV Viral load, *n* (%) Not Detected <20 copies/mL	5 (100%) 5 (100%)
Mpox PCR, *n* (%)	14 (100%)
Lesions, *n* (%) PCR lesions, *n* (%)	14 (100%) 14 (100%)
Recovery days median (range)	16 (12–26)

HIV: human immunodeficiency virus; MSM: men who have sex with men; Mpox: monkeypox; PWH: People living with HIV.

### Mpox diagnosis

Swabs from the oropharynx (TOF) and skin lesions were collected in Universal Transport Medium (UTM-RT®; COPAN Diagnostics, Italy). Whole blood samples were aliquoted using a standard EDTA blood collection tube to collect plasma. MPXV DNA was extracted with the QIAsymphony® DSP Virus/Pathogen Midi Kit/the QIAsymphony DSP DNA Mini Kit on the QIAsymphony® SP automated platform (QIAGEN, Hilden, Germany) according to the kit’s instructions. The amplification was carried out by means of real-time PCR (RT–PCR) as previously described [17]. Samples with Ct values >40 were considered negative.

### Peripheral lymphocyte isolation

Peripheral blood mononuclear cells (PBMCs) from both Mpox patients and HD were isolated by gradient centrifugation (Lympholyte, Cedarlane, Canada), counted with Trypan blue, and frozen in FBS (Fetal Bovine Serum, Euroclone, Italy) with 10% DMSO (Euroclone, Italy). Subsequently, PBMC were thawed in defrosting medium [RPMI-1640 (Corning) supplemented with 2 mmol glutamine (Gibco), 50 U/mL benzonase endonuclease (Merck)]. After washed once, PBMC were suspended at 1 × 10^6^ cells/mL in complete medium [RPMI-1640 supplemented with 10% FBS, 2 mmol glutamine, 50 IU/mL penicillin, and 50 µg/mL streptomycin (Corning)], counted by Trypan blue exclusion, and used for the experimental design.

### Phenotypic staining

The Vδ2 profile was evaluated using a dried reagent tube (DuraClone IM T cell subsets tube, Beckman Coulter, USA), containing the following antibodies: CD45RA-FITC, CCR7-PE, CD28-ECD, PD1-PC5.5, CD27-PC7, CD4-APC, CD8-A700, CD3-APCA750, CD57-Pacific Blue, and CD45-Krome Orange. We dropped in the anti-Vδ2-BV605 antibody (Biolegend). Briefly, 100 µL of PBMC suspension was loaded into the DuraClone tube and incubated for 15 min at room temperature. VersaLyse Lysing Solution (Beckman Coulter) was added and incubated for 15 min after washing cells with PBS 1X. Then, samples were washed once and fixed with paraformaldehyde 1X (PFA, Sigma). Finally, samples were acquired by flow cytometry (DxFLEX; Beckman Coulter) and analyzed with DxFlex software 2.0.2.18 (Beckman Coulter) (Supplementary Figure 1(A)).

### Intracellular staining

To evaluate Vδ2 T-cells functional properties, thawed PBMCs were stimulated with a specific phosphoantigen (PhAg: IPH1101, 3 µM; Innate-Pharma, Marseille, FR) in the presence of brefeldin A (10 µg/mL, Serva) for 18 h to allow cytokine production. The cytotoxicity of Vδ2 T-cells was analyzed by adding CD107a-APC or BV786 in all conditions overnight to analyze their cytotoxic capability. Unstimulated PBMCs were used as a negative control. The αCD3 purified antibody (1 µg/mL, Invitrogen) was used as a positive control. After the incubation, PBMC were analyzed by flow cytometry. Briefly, we stained PBMCs 15 min at 4°C with a surface anti-human monoclonal antibody cocktail: 7AAD, Vδ2-FITC, Vδ2-BV610, CD38-PE, CD38-BV610, HLA-DR-ECD, CD3-PerCP5.5, CD3-KO, TIM-3-Pacific Blue, or V450. After washed once with buffer (PBS 1×, 0.1% NaN3, 1% BSA), intracellular staining was performed with TNFα-FITC or PE and INFγ-PC7 or APC for 20 min at room temperature in the presence of permeabilizing buffer containing 0.5% saponin. Lastly, PBMCs were washed, fixed with PFA 1X, and acquired by a cytofluorimeter (Cytoflex/DxFlex, Beckman Coulter). Samples were analyzed by CyTExpert software 2.3.1.22 and DxFlex software 2.0.2.18 (Beckman Coulter). (Supplementary Figure 1(B and C)).

### Statistical analysis

The non-parametric Mann–Whitney and Wilcoxon tests assessed group differences for all variables. These statistical analyses were performed using the Graph PadPrism software 8.0. Principal Component Analysis (PCA) was performed to identify the relevant information and visualize variables with a high contribution to the immunological Vδ2 T-cell profile. Data were analyzed through R packages factoextra (for ggplot2-based visualization). To visualize a correlation matrix in R, we used the corrplot function and generated a heatmap object using correlation coefficients (computed using the Spearman correlation test) as input to the heatmap. The heatmap was produced with the R package heatmap3.

## Results

### Description of the enrolled subjects

Fourteen patients infected with Clade IIb MPXV during the 2022 outbreak were included. All were self-reported to be men who had sex with men (MSM). The median age is 41 years (32–51). Five (37.5%) were PWH, all receiving antiretroviral therapy with undetectable viremia and a median CD4 T-cell count of 670 cells/ mm^3^ (413–972). One (7.1%) received a smallpox vaccine during childhood. The median duration of the disease was 16 days (12–26); all patients successfully recovered. The main characteristics of the study population are detailed in Table 1 and Supplementary Table 1.

### Vδ2 T-cell profile and functionality at T1 and T2

**Figure 1.**
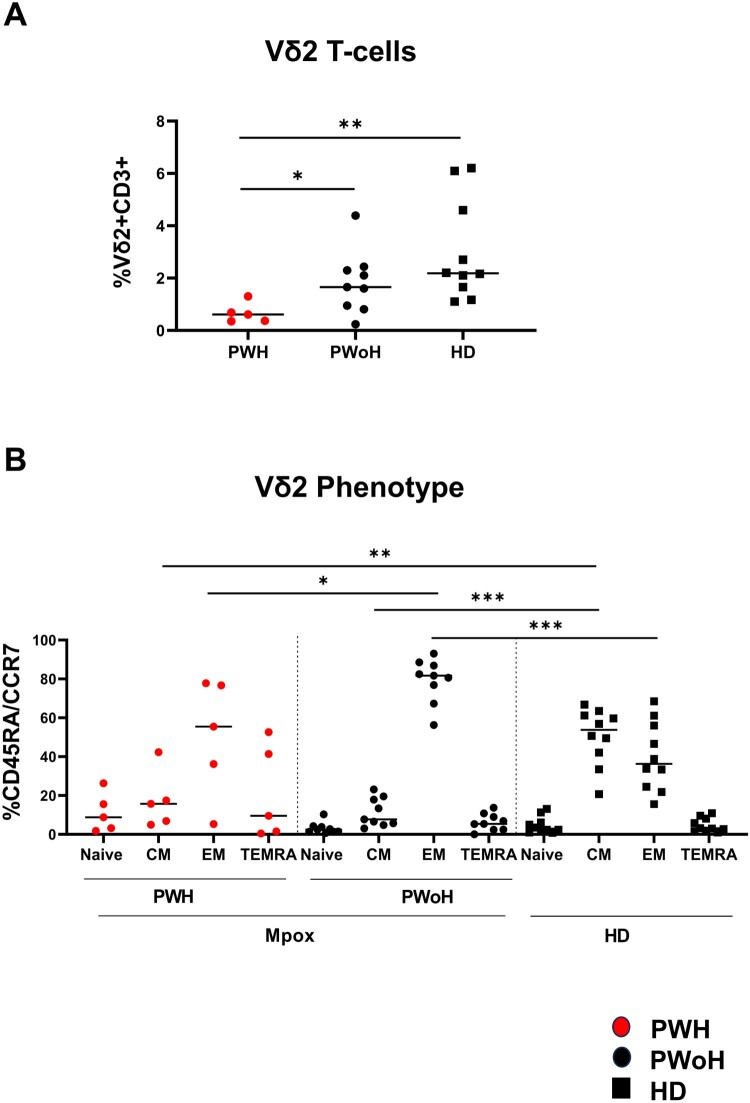
**Vδ2 T-cell phenotypic profile in the early phase of Mpox infection**. Frequency (A) differentiation profile (B) were performed on PBMC of Mpox subjects (PWH with red dots and PWoH with black dots) and healthy controls (black square) by multiparametric flow cytometry in the early phase of Mpox infection (T1). Statistical significance was performed by GraphPad Prism. (A) *p = 0.02; **p = 0.0027. (B) *p = 0.01; **p = 0.004; ***p = 0.0001.

In the acute phase (T1), Vδ2 T-cells were lower in PWH patients relative to PWoH (people living without HIV) (*p *= 0.02) and to HD (*p *= 0.0027) (Figure 1(A)). As shown in Figure 1(B), PWH patients had a lower frequency of effector memory cells (*p *= 0.01) compared to PWoH (*p *= 0.01). Both groups showed a lower frequency of central memory cells (PWH *p *= 0.004; PWoH *p *= 0.0001) with respect to HD. Finally, PWoH showed a higher frequency of effector memory cells (*p *= 0.0001) compared with HD.

**Figure 2.**
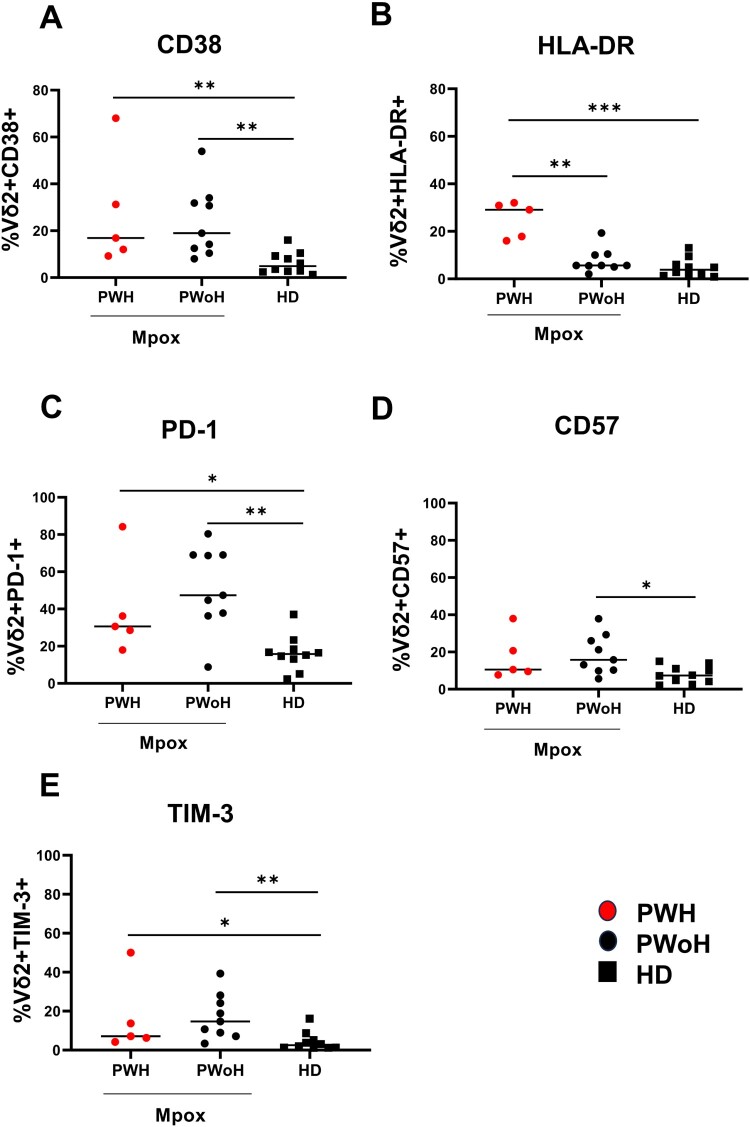
**Vδ2 T-cell activation/exhaustion markers in the early phase of Mpox infection**. Activation markers (A–B: CD38 and HLA-DR), exhaustion and senescence markers (C–E: PD-1, CD57, TIM-3) were performed on PBMC of Mpox subjects (PWH with red dots and PWoH with black dots) and healthy controls (black square) by multiparametric flow cytometry in the early phase of Mpox infection (T1). Statistical significance was performed by GraphPad Prism. (A) **p = 0.005; **p = 0.001. (B) ***p = 0.0007; **p = 0.003. (C) *p = 0.01; **p = 0.002. (D) *p = 0.01; (E) *p = 0.04; **p = 0.002.

**Figure 3.**
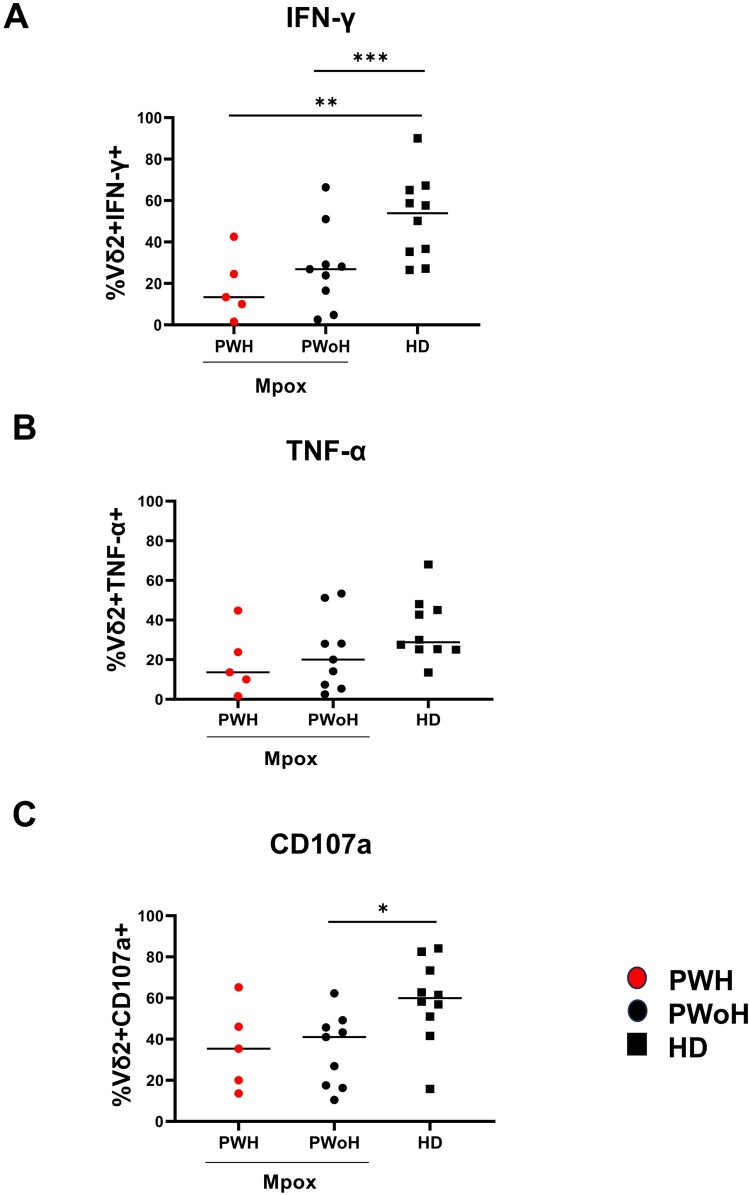
**Vδ2 T-cell functionality in the early phase of Mpox infection**. IFN-γ (A) and TNF-α (B) production, as well as CD107a (C) expression, were performed by multiparametric flow cytometry on PBMC of Mpox subjects (PWH with red dots and PWoH with black dots) and healthy controls (black square) after 20 h of specific PhAg stimulation in the early phase of Mpox infection (T1). Statistical significance was performed by GraphPad Prism. (A) *p = 0.02; **p = 0.008. (C) *p = 0.01.

Vδ2 T-cells of patients showed a higher expression of CD38 (PWH *p *= 0.005; PWoH *p *= 0.001) and HLA-DR [(PWH *p *= 0.003; PWoH *p *= 0.0007) Figure 2(A and B))] activation markers. A higher expression of PD-1 (PWH *p *= 0.01; PWoH *p *= 0.002), dfure 2(C) and CD57 (PWoH *p *= 0.01), Figure 2(D) exhaustion/senescence markers, along with the checkpoint inhibitory marker TIM-3 (PWH *p *= 0.04; PWoH *p *= 0.002), Figure 2(E) was also observed on Vδ2 T-cells of patients relative to those of HD. Based on this profile, we analyzed the Vδ2 T-cell functionality by stimulating the PBMC with a Vδ2-specific PhAg. The results showed that IFN-γ-producing Vδ2 T-cells (PWH *p *= 0.008; PWoH *p *= 0.02), Figure 3(A) and CD107a expression on Vδ2 T-cells (PWoH *p *= 0.01, Figure 3(C)) were lower in patients relative to HD. Vδ2 T-cells functionality in experimental controls (Unstimulated or UNS and aCD3-stimulated PBMC) was reported as follows: Mpox (Vδ2/IFNγ UNS vs. PhAg *p *= 0.0002; Vδ2/IFNγ UNS vs. aCD3 *p *= 0.0002; Vδ2/CD107a UNS vs. PhAg *p *= 0.0002; Vδ2/CD107a UNS vs. aCD3 *p *= 0.0002) and HD (Vδ2/IFNγ UNS vs. PhAg *p *= 0.01; Vδ2/IFNγ UNS vs. aCD3 *p *= 0.03; Vδ2/CD107a UNS vs. PhAg *p *= 0.07; Vδ2/CD107a UNS vs. aCD3 *p *= 0.01). No differences were observed in TNFα production with respect to HD (PWH *p *= 0.055; PWoH *p *= 0.24, Figure 3(B)). Interestingly, the altered IFNγ/Vδ2 functionality was inversely correlated with activation markers expression of CD38 (*r* = −0.55, *p *= 0.005) and HLA-DR (*r* = −0.58; *p *= 0.01), with TIM-3 (*r* = −0.4; *p *= 0.04) and PD-1 expression (*r* = −0.5; *p *= 0.01), Supplementary Figure 2(A–D), and not with Mpox Viral Load or recovery days (*p *= 0.49 and *p *= 0.99, respectively). HIV infection affected Vδ2 T-cell frequency, effector memory profile and HLA-DR expression in co-infected subjects.

At T2, 10/14 subjects returned to the hospital for blood draw. We observed that Vδ2 T-cells prolonging their activated/exhausted profile 20 days upon symptom onset even if the median of recovery is 16 days (range 12–26). Specifically, a continuous perturbation of Vδ2-markers was seen in patients relative to HD, substantiating the prolonged effect of the Mpox infection on Vδ2 T-cells also after recovery. In particular, TIM-3 expression showed a trend of decrease at T2 significant with respect to HD (*p *= 0.008) (Figure 6(D)). These data show a highly activated Vδ2-profile with reduced functionality in the first phases of Mpox infection, underlining their possible role in resolving the infection.

### Principal component analysis of Mpox patients at T1

**Figure 4.**
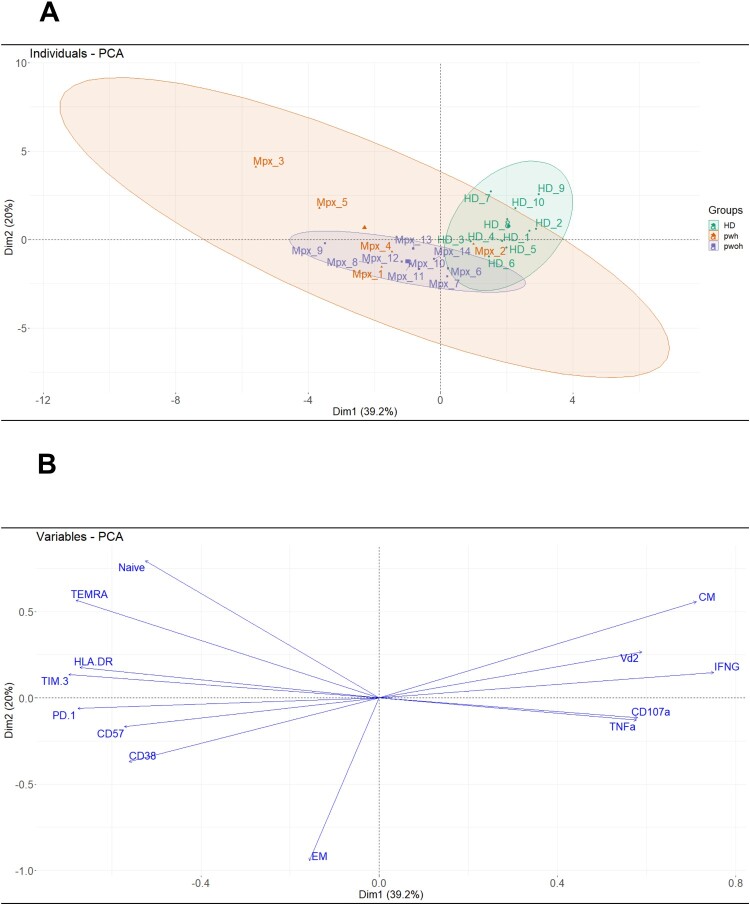
**Vδ2 T-cell profile in the acute phase of Mpox infection.** Principal Component Analysis of Vδ2 T cell profile in Mpox subjects (early phase of infection, T1) and in healthy controls, marked in orange and green (A), respectively, and the contributing variables plot (B). The cumulative variance of PCs 1 and 2 was 59.2%, while for each sample group, 95% confidence ellipses and centroids were calculated.

To identify a possible specific Vδ2 T-cell innate profile defining the acute phase of Mpox infection, we performed a PCA. The results of this analysis showed a clear and differentiated distribution of both groups, Mpox and HD (Figure 4(A)), along with a high cumulative variance (PC1 39.2% and PC2 20%, Supplementary Figure 3), reflecting the segregation of analyzed samples due to the higher contribution of specific variables (Figure 4(B)). These data confirmed that the altered and impaired Vδ2 T-cell profile of patients was relayed to exhaustion/activation marker expression and to an effector phenotype. On the contrary, the Vδ2 T-cell profile of HD was associated with a functional naive and CM phenotype, producing antiviral and proinflammatory cytokines. We performed a heat map analysis in order to evaluate the contribution of each marker in this specific Vδ2 profile (Supplementary Figure 4(A and B)). In patients, a significant association of Vδ2 T-cell frequency with the expression of naïve (*r* = −0.7, *p *= 0.001), effector memory (*r* = 0.7, *p *= 0.001), TEMRA cells (*r* = 0.6, *p *= 0.02) and exhaustion/inhibitory markers (CD57: *r* = 0.7, *p *= 0.004 and CD38/TIM-3: *r* = 0.6, *p *= 0.01) was observed. Instead, in HD Vδ2 T-cells, were associated with the expression of naïve (*r* = 0.7, *p *= 0.01) and central memory cells (*r* = 0.7, *p *= 0.009) not activated (CD38: *r* = 0.6, *p *= 0.03) with a functional profile (IFN-γ: *r* = −0.7, *p *= 0.02).

### Vδ2 T-cell profile and functionality at T3M

**Figure 5.**
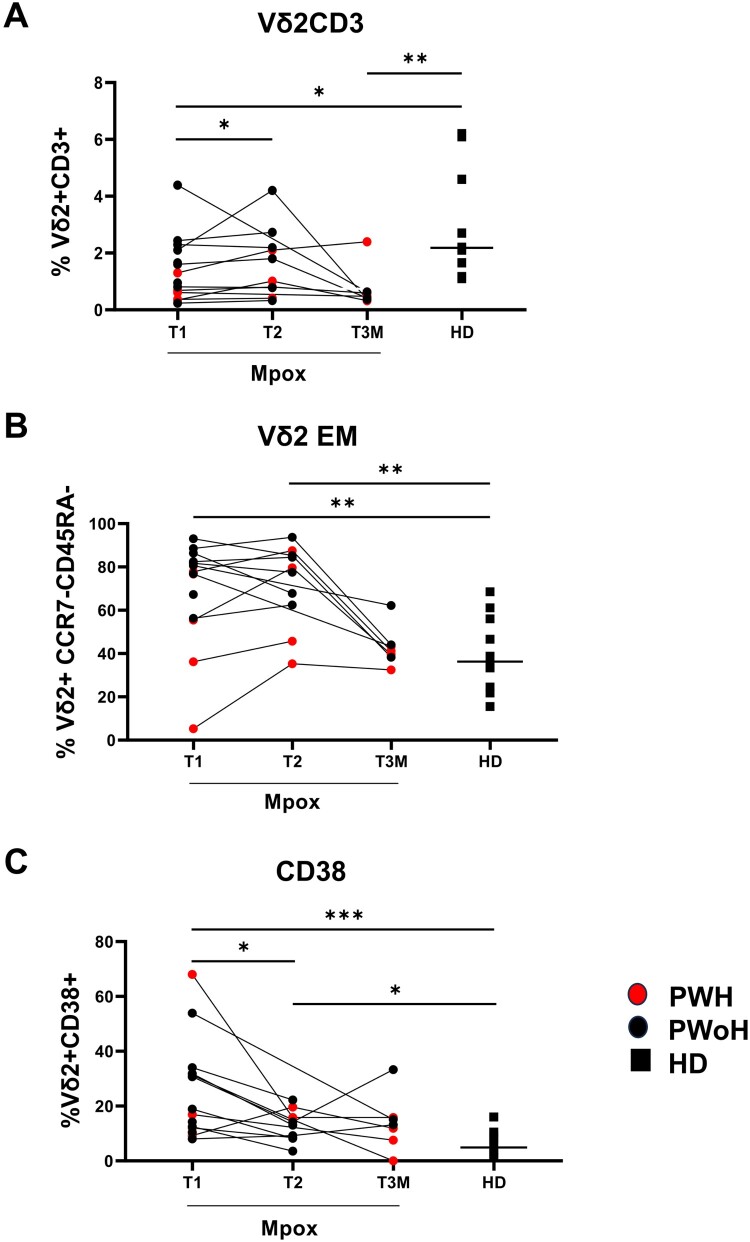
**Vδ2 T-cell phenotypic profile after the acute phase up to three months from infection**. Longitudinal analysis of the frequency (A), differentiation profile (B) and CD38 expression were performed on PBMC of Mpox subjects (PWH with red dots and PWoH with black dots) and healthy controls (with black square dots and median value was shown as a line) by multiparametric flow cytometry at T2 and T3M post infection. Statistical significance was performed by GraphPad Prism. (A) T1 vs T2: *p = 0.01; T1 vs HD: *p = 0.01; T3M vs HD: **p = 0.003. (B) T1 vs HD: **p = 0.003; T2 vs HD: **p = 0.002. (C) T1 vs T2: *p = 0.01; T1 vs HD: ***p = 0.0002; T2 vs HD: *p = 0.01.

**Figure 6.**
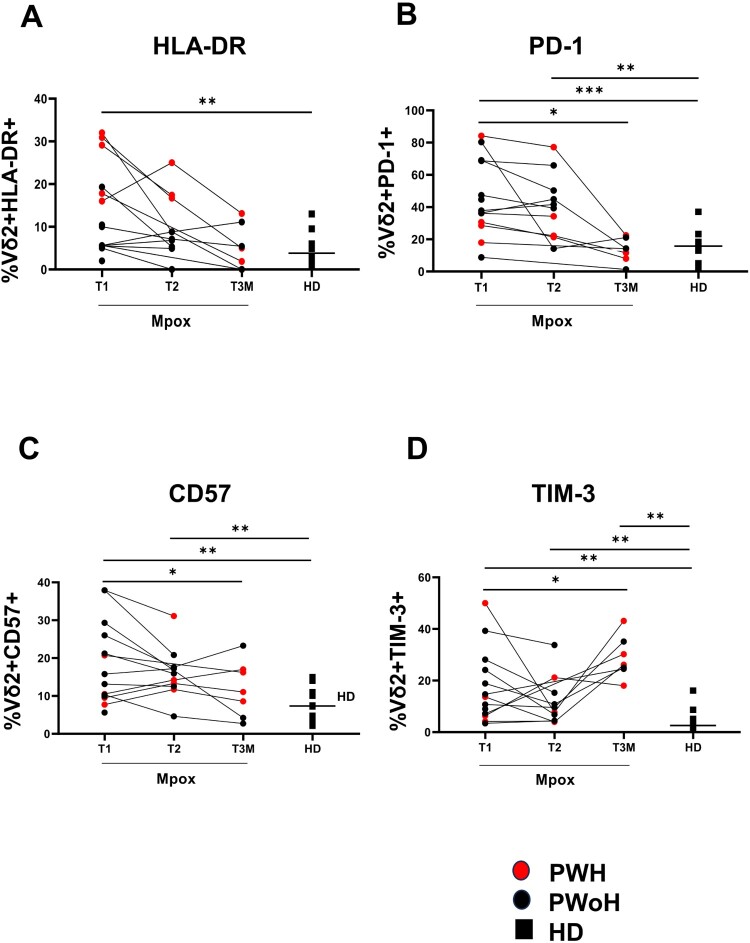
**Vδ2 T-cell activation/exhaustion markers after the acute phase up to three months from infection**. Activation marker (HLA-DR), exhaustion and senescence markers (B-D: PD-1, CD57, TIM-3) were performed on PBMC of Mpox subjects (PWH with red dots and PWoH with black dots) and healthy controls (with black square dots and median value was shown as a line) by multiparametric flow cytometry at T2 and T3M post infection. Statistical significance was performed by GraphPad Prism. (A) T1 vs HD: **p = 0.005. (B) T1 vs T3M: *p = 0.01; T1 vs HD: ***p = 0.0007; T2 vs HD: **p = 0.002. (C) T1 vs T3M: *p = 0.01; T1 vs HD: **p = 0.008; T2 vs HD: **p = 0.005. (D) T1 vs T3M: *p = 0.01; T1 vs HD: **p = 0.001; T2 vs HD: **p = 0.008; T3 vs HD: **p = 0.001.

**Figure 7.**
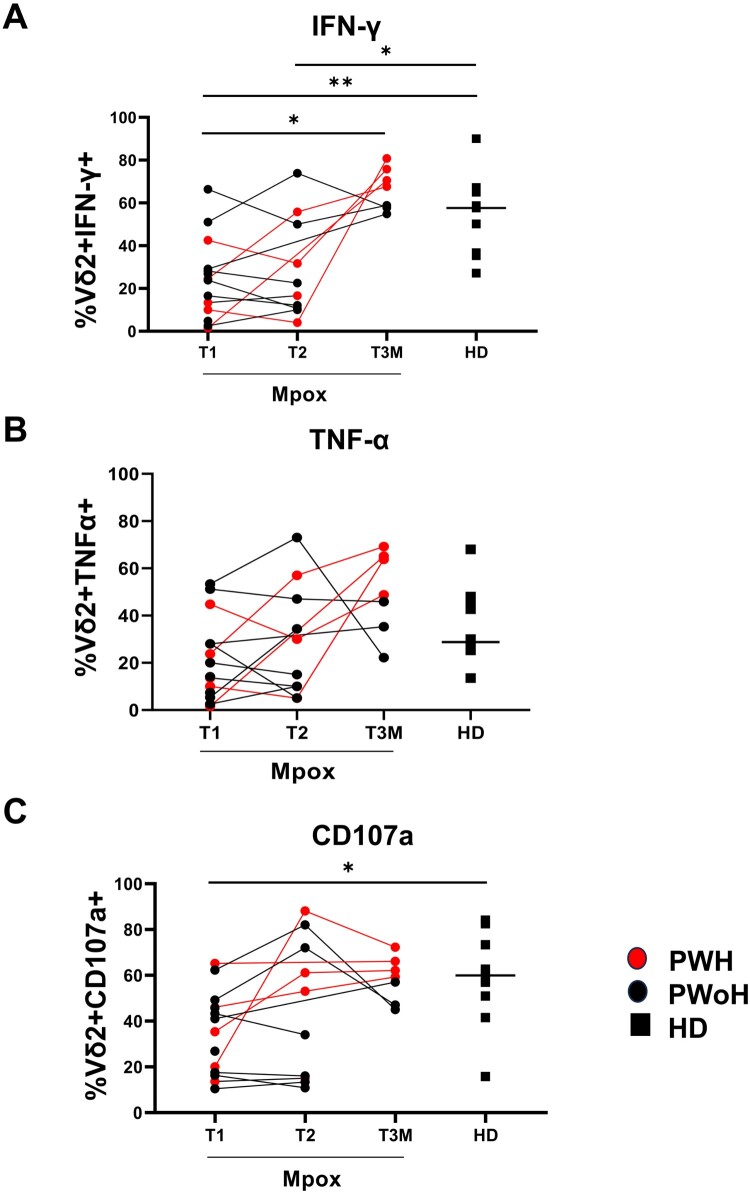
**Vδ2 T-cell functionality after the acute phase up to three months from Mpox infection**. IFN γ (A) and TNFα (B) production, as well as CD107a expression (C), were performed by multiparametric flow cytometry on PBMC of Mpox subjects (PWH with red dots and PWoH with black dots) and healthy controls (with black square dots and median value was shown as a line) after 18 h of specific PhAg stimulation at T2 and T3M post infection. Statistical significance was performed by GraphPad Prism. (A) T1 vs T3M: *p = 0.04; T1 vs HD: **p = 0.038; T3M vs HD: *p = 0.02. (C) T1 vs HD: *p = 0.01.

At T3M, 7/14 patients returned for blood draw. The Vδ2 T-cells frequency was lower in patients relative to HD (*p *= 0.003, Figure 5(A)), while effector memory cells (*p *= 0.84, Figure 5(B)), markers of activation (CD38: *p *= 0.10, Figure 5(C)); and HLA-DR: *p *= 0.93, Figure 6(A), of functional exhaustion (PD-1: *p* = 0.53, Figure 6(B)) and senescence (CD57: *p *= 0.053, Figure 6(C)), returned to HD values. In addition, TIM-3 expression showed a higher expression related to HD (*p *= 0.001, Figure 6(D)). Unlike the acute infection, there were no associations between the levels of IFN-γ and frequency of CD38 (*r* = −0.07, *p *= 0.77), HLA-DR (*r* = −0.26; *p *= 0.29) and TIM-3 (*r* = 0.38; *p *= 0.12), while a negative correlation was observed with PD-1 expression considering all Mpox patients’ timepoints (R: −0.5, *p *= 0.001, Supplementary Figure 5). Furthermore, a complete recovery of Vδ2 T-cell functionality was observed, as shown by IFN-γ (*p *= 0.13) TNFα (*p *= 0.98) and CD107α (*p *= 0.41) levels similar to those of HD (Figure 7(A–C)) paralleled to patients’ recovery. Additionally, Vδ2 T-cell cytokines production was higher in PWH (Vδ2 /IFN-γ *p *= 0.02; Vδ2/TNF-α *p *= 0.01) when compared with PWoH (Figure 7(A and B)). Three months post-infection data indicated a significant restoration of Vδ2 lymphocyte profile and functionality in both PWH and PWoH, correlating with complete patient recovery.

## Discussion

We provide the first *in vivo* demonstration that changes in the functional profile of circulating γδ T-cells occur in the context of human MPXV infection. Specifically, our data demonstrate that in the acute phase of Mpox, e.g. a few days post-infection, MPXV interferes with Vδ2 T-cell functionality by impairing the production of IFN-γ after PhAg stimulation. This impaired IFN-γ production is linked to a hyperexpression of activation (CD38 and HLA-DR) and inhibitory (TIM-3 and PD-1) markers on Vδ2 T-cell surface. No correlation was observed with Mpox viral Load or recovery days. As early as three months post-infection, Vδ2 T-cells restore their effector functions, likely due to a TIM-3-independent pathway as observed for the natural killer (NK) cells [18,19].

Here, we discuss our findings in the context of current literature. We address the potential clinical and biological implications of our results and conclude by presenting our study limitations and future directions for expanding our work.

Our data demonstrate that in the acute phase of Mpox infection, Vδ2 T-cells undergo an alteration of their frequency and profile by increasing the expression of activation (CD38 and HLA-DR), exhaustion/ senescence (PD-1 and CD57), and inhibitory markers (TIM-3). These changes are similar in PWH and PWoH with the exception of a decrease in Vδ2 frequency and effector profile as well as an increase in HLA-DR expression and functionality in PWH with respect to PWoH. Our data are in line with our previous observation on the involvement of adaptive T-cell response during the acute phase of Mpox infection [20]. In fact, CD4 and CD8 T-cells play an essential role in eradicating MPXV, with an engagement of MPXV-specific αβ T-cell response *in vivo* during the early stages of infection, contributing to clearing the virus from the host [20,21]. The involvement of the innate immune system activation is in line with data related to several infections. In the Poxviruses infection, a strong activation of NK cells in the respiratory illness in mice from the Vaccinia virus is seen [22], suggesting that their recruitment and activation could contribute to the lung’s protection. The evidence of a potent soluble Vδ2-antiviral activity against the Vaccinia virus *in vitro* [15] and the Orthopoxviruses *in vivo*-expansion and recall of effector Vδ2 T-cells in a macaque model [16] defined the important role of IFN-γ-producing γδ T-cells in innate resistance to Poxviruses. This evidence also sheds light on our understanding of the protective type 1 γδ memory immunity in the host. Furthermore, an important antiviral role of the γδ T-cells was recognized in VV-infected mice *in vivo*. A strong and rapid γδ innate expansion and response against VV infection in both normal C57BL/6 mice and beta TCR knock-out (KO) mice were observed [23]. A similar NK cytotoxic function was observed in this model, showing that in KO mice, γδ T-cells have a constitutive cytolytic function associated with a peak of γδ T-cell response at day 8 from infection, decreasing in number after that. Thus, γδ T-cells may be mediators of innate immunity against Poxviruses, significantly impacting the early stages of virus replication in the absence of an adaptive response [23]. Recent data also show the involvement of resident γδ T-cells in response to cutaneous Vaccinia virus infection, where they control and clear the virus collaborating with circulating Vδ2 T cells [24]. In this context, γδ T-cells can contribute to wound healing after clearing the virus via the production of multiple cytokines/growth factors, modulation of tissue response, and maintenance of a skin barrier to prevent a secondary infection. It was also reported that there was an inhibition of γδ T-cell functionality *in vitro* by Orthopoxviruses [25]. Similarly, our data on the Vδ2 T-cell functional impairment a few days after Mpox infection underscore their involvement against the MPXV and demonstrate their ability to clear the virus along with other players of innate and adaptive immunity. This altered and dysfunctional Vδ2 profile, prolonged after the patient’s recovery and followed by functional exhaustion, was observed in infections caused by other emerging viruses [26], such as the Dengue infection [27]. In literature, the expression of exhaustion and inhibitory receptors, such as PD-1 and TIM-3, is linked to a lack of functionality on T-cells during viral infections [28] and cancer [29]. In our model, we observed a similar inhibiting effect of Mpox infection on Vδ2 T-cell response in the acute phase of infection and after patients’ recovery. γδ T-cell response inhibition in the context of Poxvirus infection may be a host-evading mechanism and a pathogenic virus-dependent pathway in the host.

A limitation of our study is the low number of enrolled patients, few of whom had blood drawn at T2 and T3M. Notwithstanding this consideration, we believe that the biological impact of the data obtained in this study shows clearly the effects of MPXV on Vδ2 T-cell profile in the early stages of infection, exhibiting their capability to produce pro-inflammatory antiviral cytokines and cytotoxic factors able to clear the virus, although their exhausted profile. These specific Vδ2 T-cell properties highlight their role in the protection/pathogenesis mechanisms of preventing Mpox severe infection. Finally, future studies correlating our biological findings with clinical outcomes in larger cohorts are ensured, as well as the involvement of Vδ2 T-cell direct antiviral mechanisms during Mpox infection.

## Supplementary Material

Cimini E Supplements.pdf
