# Supplementary material for: Vδ2 T-cells response in people with Mpox infection: a three-month longitudinal assessment
Source: Emerg Microbes Infect. 2025 Jan 16;14(1):2455585. doi: 10.1080/22221751.2025.2455585 (PMC11792154; doi:10.1080/22221751.2025.2455585)

Supplements

Supplementary Table 1. MPXV Viral load of the study cohort at T1.

	Cutaneous lesion (ct)	Plasma (ct)	TOF (ct)
Mpox1	15.16	32.14	23.92
Mpox2	18.54	36.53	NA
Mpox3	20.50	31.63	NA
Mpox4	18.13	NA	NA
Mpox5	20.00	35.00	Negative
Mpox6	20.11	Negative	35.40
Mpox7	13.64	35.52	30.33
Mpox8	14.37	32.79	21.64
Mpox9	21.43	31.56	29.58
Mpox10	33.64	30.88	NA
Mpox11	26.39	30.10	27.35
Mpox12	15.00	NA	Negative
Mpox13	16.00	NA	29.00
Mpox14	17.00	NA	37.00

NA: not available

Supplementary Figure 1. Vδ2 T-cells gating strategy.

A) Representative dot plots for identifying Vδ2 T-cells for healthy donors and all enrolled patients: doublets were excluded in the FSC-H and FSC-A dot plots. In the immunological plot (SSC-A and CD45), the CD45+ cells were gated as lymphocytes, followed by gating on CD3+ cells. **B)** Vδ2+ T-cells were then selected in CD3+, and the expression of differentiation profile (CD45RA and CCR7), activation (CD38), exhaustion (PD-1) and senescence marker (CD57) was analyzed in the Vδ2 T-cells population as well as **C)** the cytokine's production (IFN-γ and TNF-α) and CD107a expression in live cells.

A) Gating strategy for Vδ2 T-cells immunophenotyping.

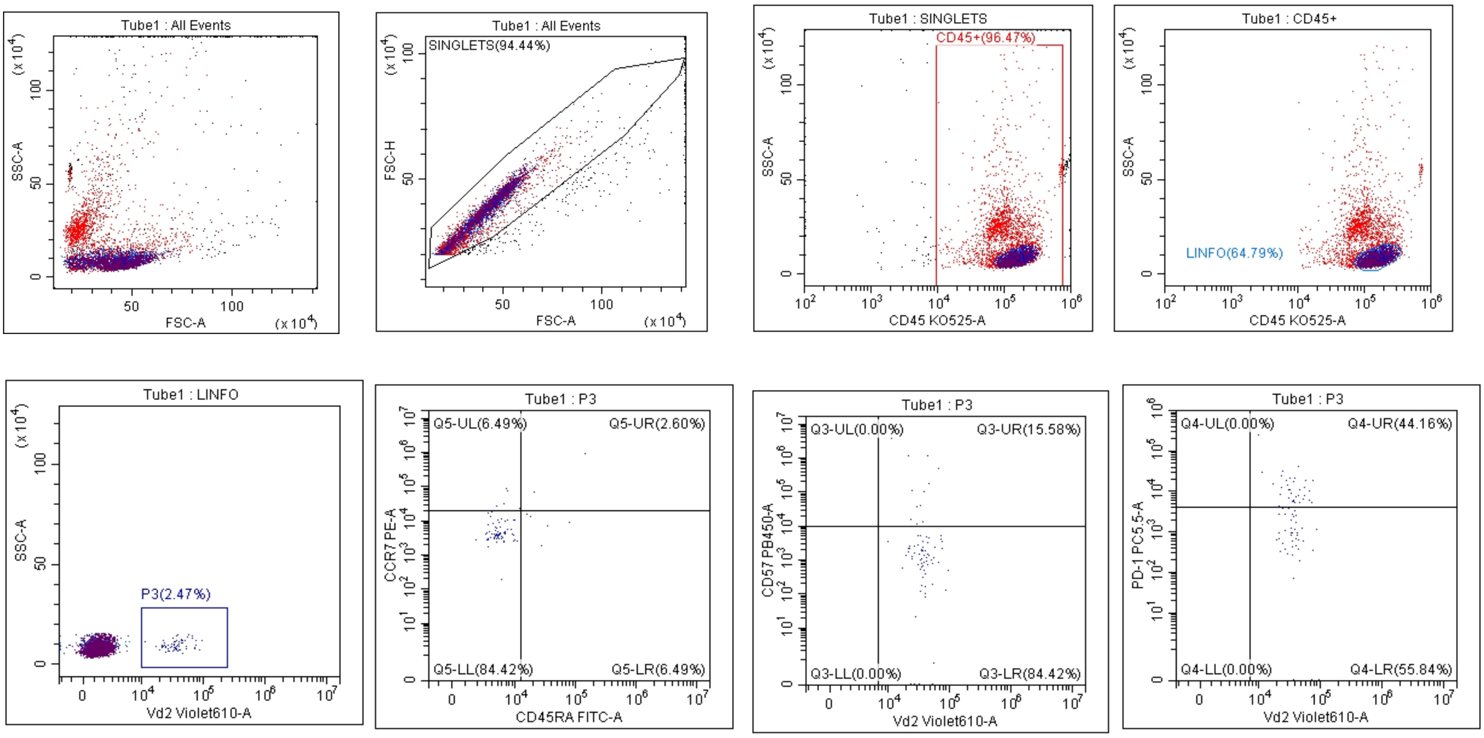

B) Gating strategy for Vδ2 T-cells markers.

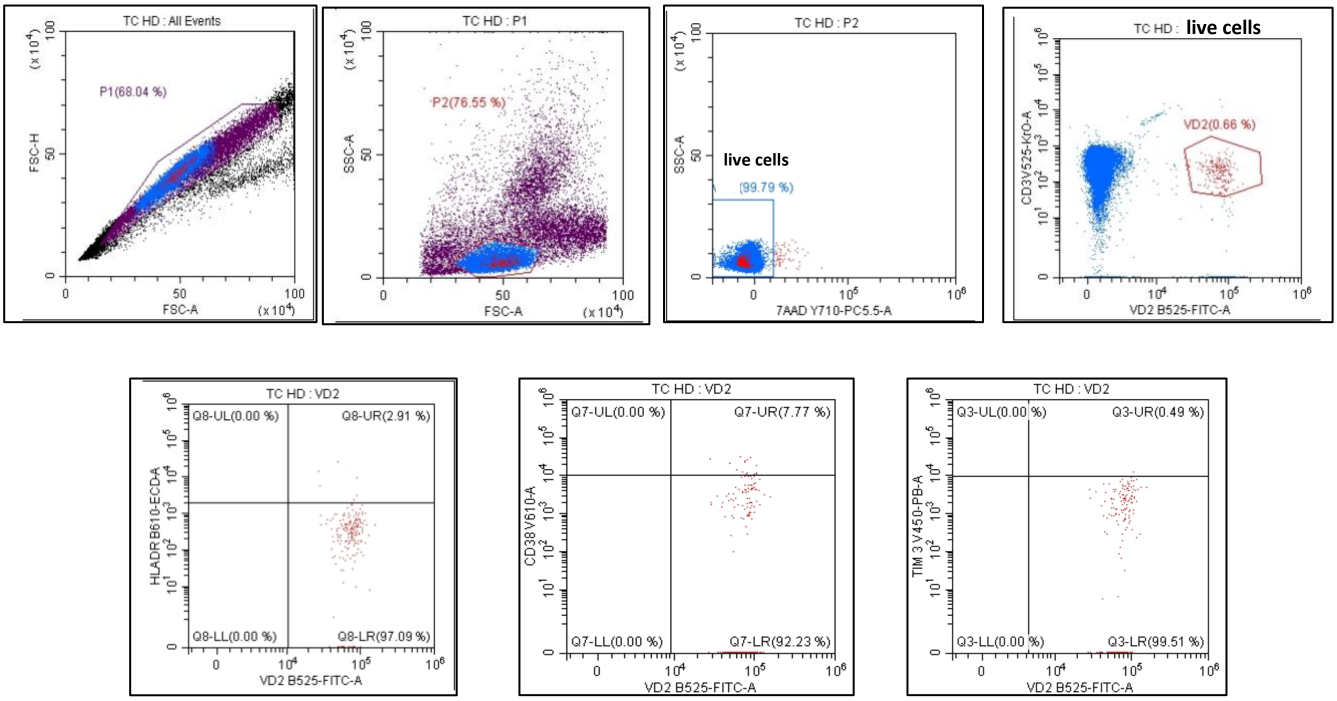

C) Gating strategy for Vδ2 T-cells functionality.

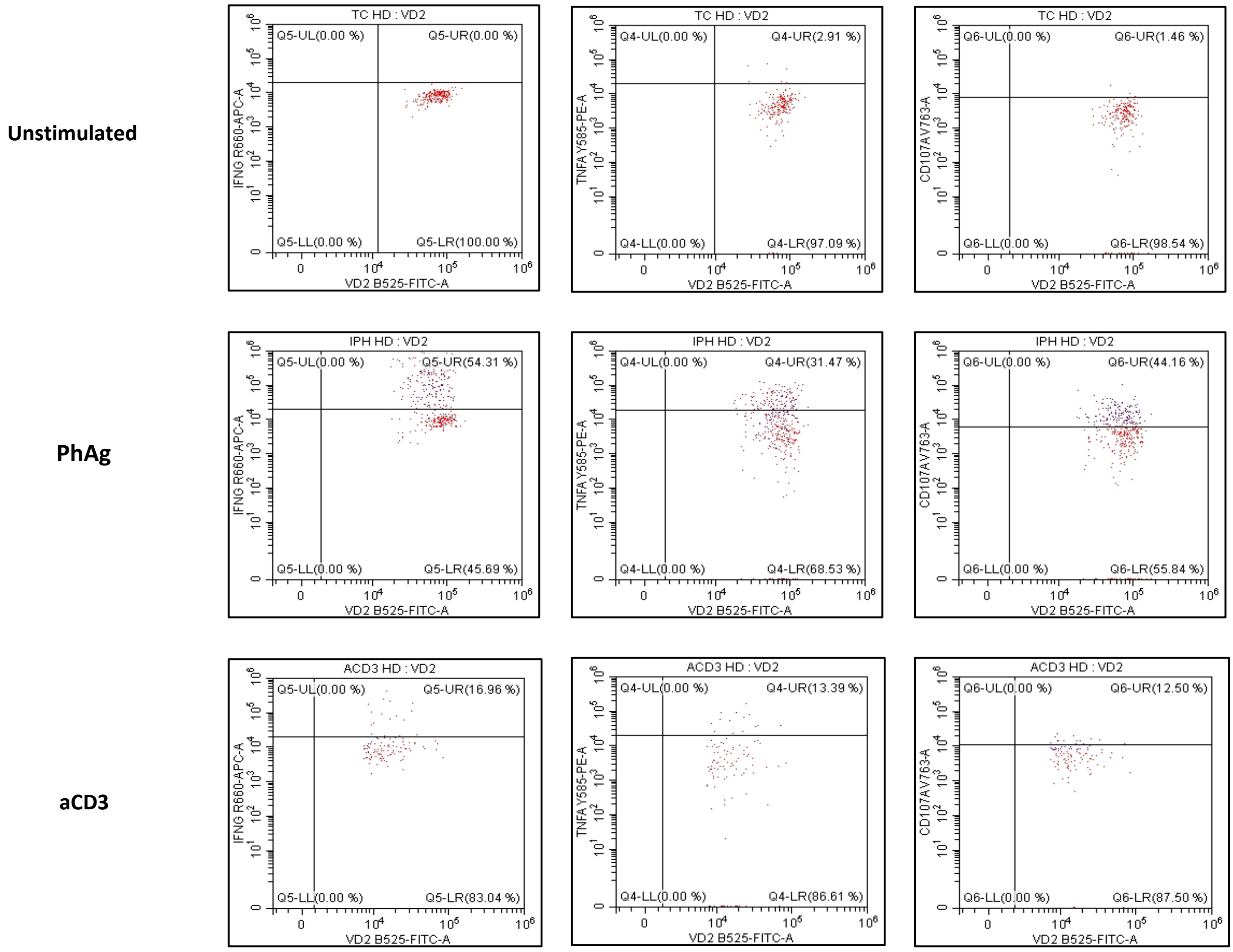

Supplementary Figure 2. **IFNγ-Vδ2-producing T-cells correlation with activation and exhaustion markers.** The correlation of significative analyzed markers with IFNγ- producing-Vδ2 T-cells was reported in HD and Mpox subjects at the early phase of infection (T1), (panel A: R: -0.5 and P=0.005; panel B: R: -0.5 and P=0.01; Panel C: R: -0.4 and P=0.04; Panel D: R: -0.5 and P=0.01).

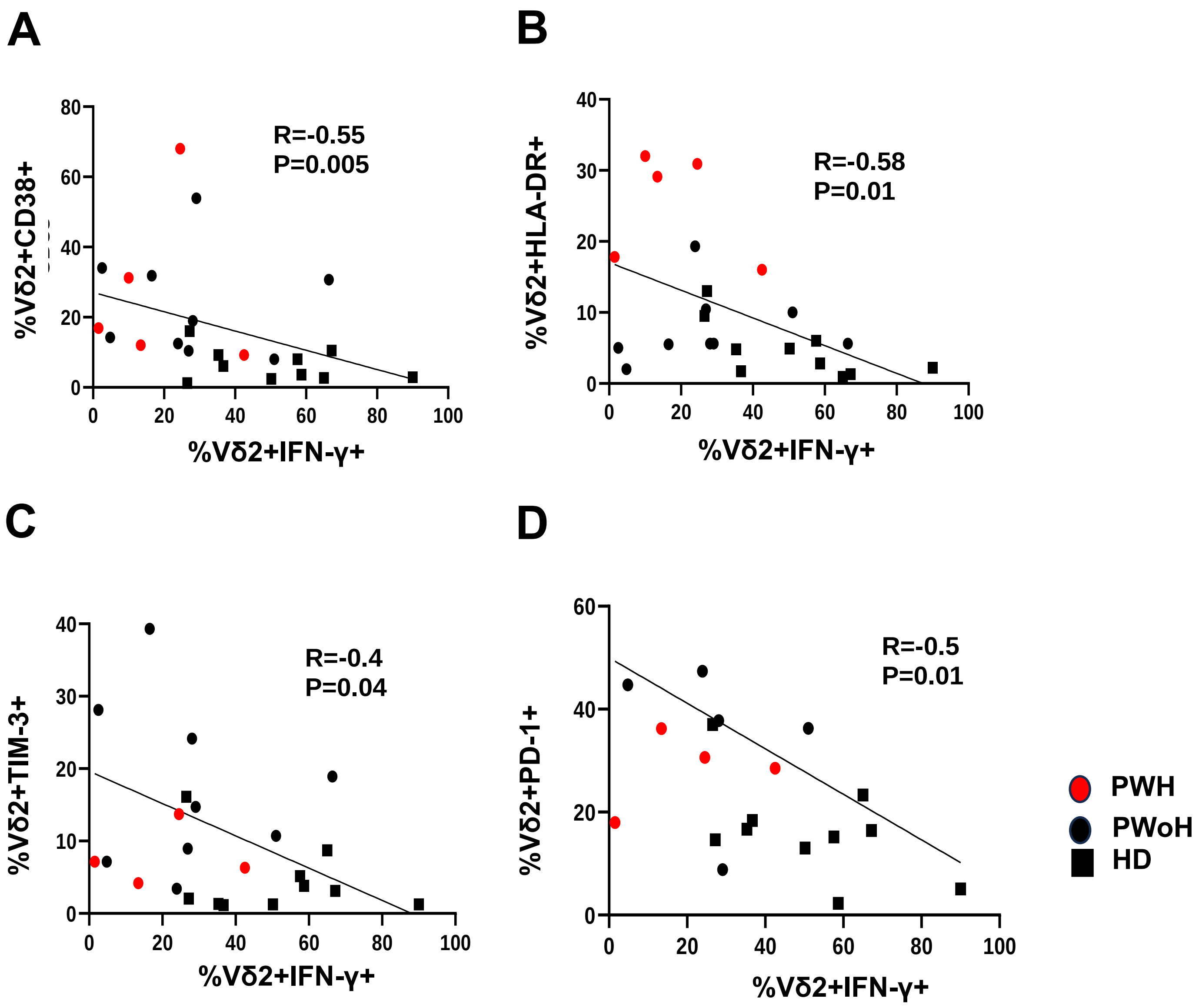

Supplementary Figure 3. **Percentage of explained variance**. The explained variance for all 13 PCs was linked to PCA analysis. The graphic showed that the cumulative variance for first two PCs is 59.2%.

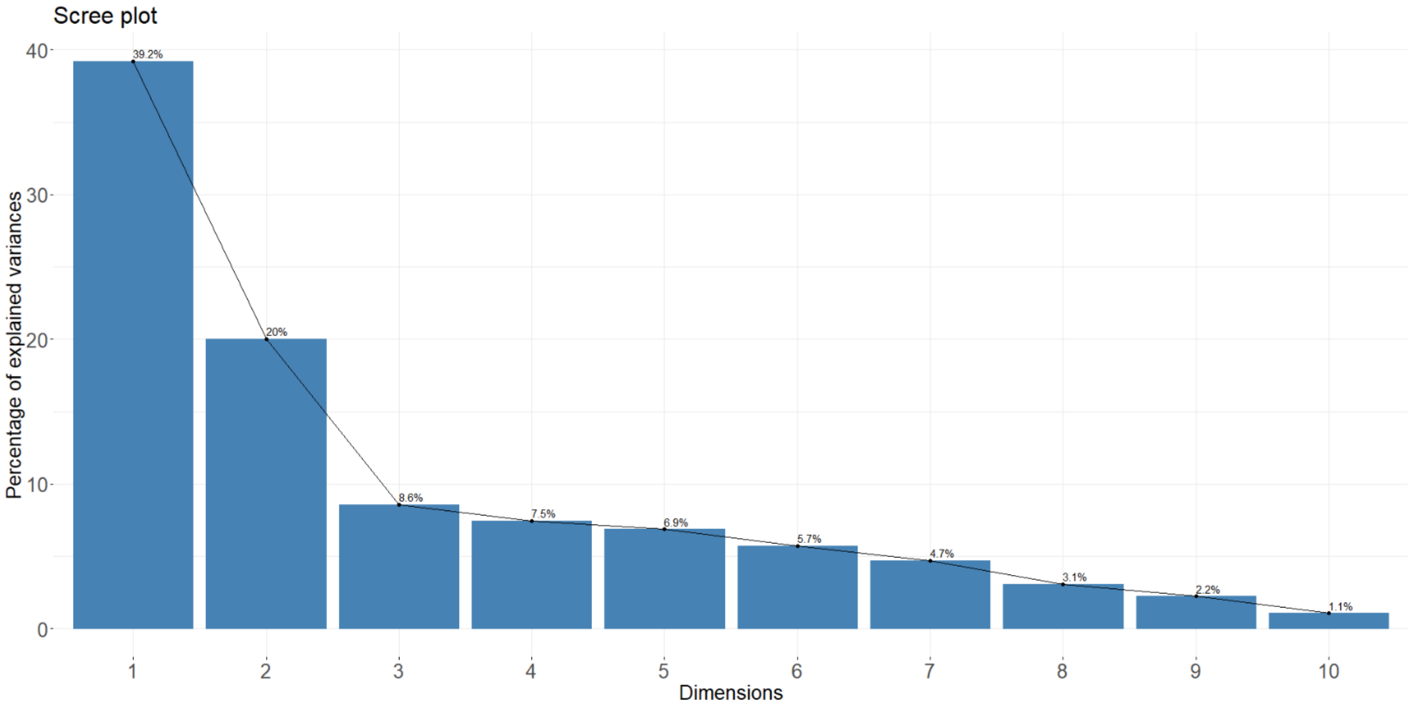

Supplementary Figure 4. **Heat map analysis of Vδ2 T-cells profile in HD and in the early phase of Mpox infection**.

The heat map analysis depicts the correlation between all variables investigated from Mpox subjects in the early phase of infection (T1) and in controls. Brighter colors (e.g., red) indicate a stronger negative correlation, while darker colors (e.g., blue) indicate a stronger positive correlation. Spearman correlation test was used, white circles showed R number and significant values. Briefly in Vδ2/CD3 T cells of HD (Panel A): Vδ2/Naïve p=0.017; CM: p=0.009; Vδ2/EM: p=0.009; Vδ2/CD38: p=0.031; Vδ2 Naïve/Vδ2 EM p=0.0001; Vδ2 Naïve/Vδ2 CD38 p= 0.049; Vδ2 CM/Vδ2 EM p=0.0001; Vδ2 EM/ Vδ2 CD38 p=0.05; Vδ2 HLA-DR/ Vδ2 IFNγ p=0.023. In Mpox subjects (Panel B): Vδ2/Naïve p=0.001; Vδ2/EM: p=0.001; Vδ2/ TNFα: p=0.02;Vδ2 Naïve/Vδ2 EM p=0.002; Vδ2 Naïve/Vδ2 TEMRA p=0.02; Vδ2 Naïve / Vδ2 IFNγ p= 0.002; Vδ2 Naïve / Vδ2 TNFα p= 0.006; Vδ2 CM/ Vδ2 EM p=0.01; Vδ2 EM/Vd2 IFNγ p= 0.054; Vδ2 EM/ Vδ2 TNFα p= 0.059; Vδ2 TEMRA /Vd2 CD57 p= 0.004; Vδ2 TEMRA/Vd2 IFNγ p= 0.04; Vδ2 CD38/ Vδ2 TIM-3 p=0.01; Vδ2 IFNγ / Vδ2 TNFα p= 0.0005.

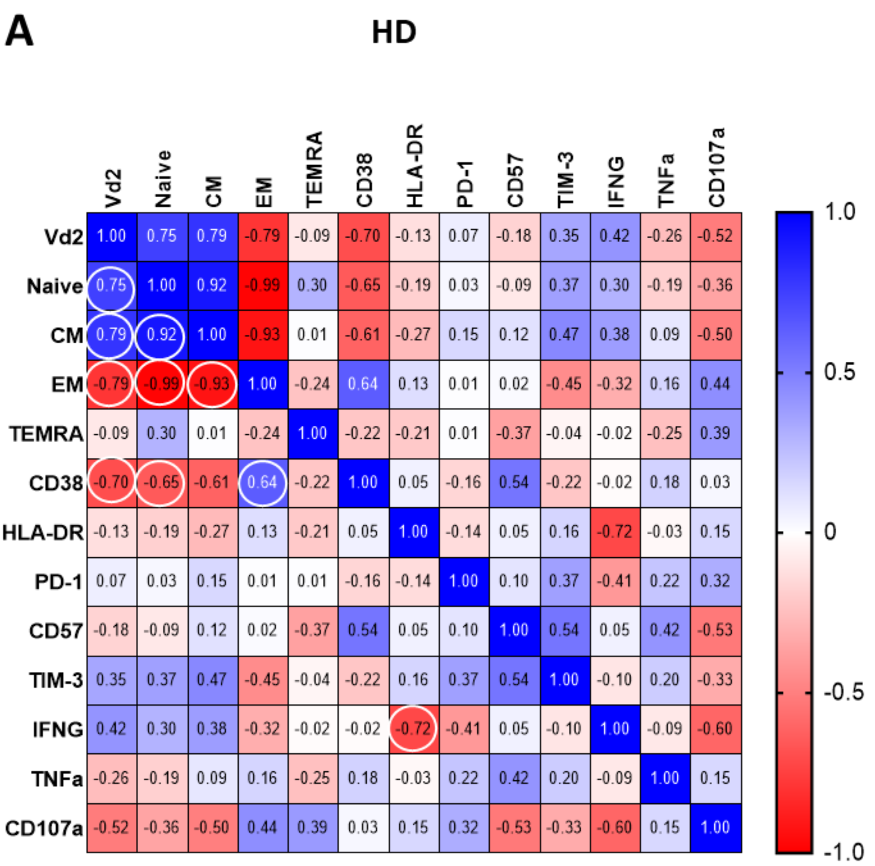

	Vd2	Naive	CM	EM	TEMRA	CD38	HLA-DR	PD-1	CD57	TIM-3	IFNG	TNFa	CD107a
Vd2	1.00	0.75	0.79	-0.79	-0.09	-0.70	-0.13	0.07	-0.18	0.35	0.42	-0.26	-0.52
Naive	0.75	1.00	0.92	-0.99	0.30	-0.65	-0.19	0.03	-0.09	0.37	0.30	-0.19	-0.36
CM	0.79	0.92	1.00	-0.93	0.01	-0.61	-0.27	0.15	0.12	0.47	0.38	0.09	-0.50
EM	-0.79	-0.99	-0.93	1.00	-0.24	0.64	0.13	0.01	0.02	-0.45	-0.32	0.16	0.44
TEMRA	-0.09	0.30	0.01	-0.24	1.00	-0.22	-0.21	0.01	-0.37	-0.04	-0.02	-0.25	0.39
CD38	-0.70	-0.65	-0.61	0.64	-0.22	1.00	0.05	-0.16	0.54	-0.22	-0.02	0.18	0.03
HLA-DR	-0.13	-0.19	-0.27	0.13	-0.21	0.05	1.00	-0.14	0.05	0.16	-0.72	-0.03	0.15
PD-1	0.07	0.03	0.15	0.01	0.01	-0.16	-0.14	1.00	0.10	0.37	-0.41	0.22	0.32
CD57	-0.18	-0.09	0.12	0.02	-0.37	0.54	0.05	0.10	1.00	0.54	0.05	0.42	-0.53
TIM-3	0.35	0.37	0.47	-0.45	-0.04	-0.22	0.16	0.37	0.54	1.00	-0.10	0.20	-0.33
IFNG	0.42	0.30	0.38	-0.32	-0.02	-0.02	-0.72	-0.41	0.05	-0.10	1.00	-0.09	-0.60
TNFa	-0.26	-0.19	0.09	0.16	-0.25	0.18	-0.03	0.22	0.42	0.20	-0.09	1.00	0.15
CD107a	-0.52	-0.36	-0.50	0.44	0.39	0.03	0.15	0.32	-0.53	-0.33	-0.60	0.15	1.00

B **Mpox early phase (T1)**

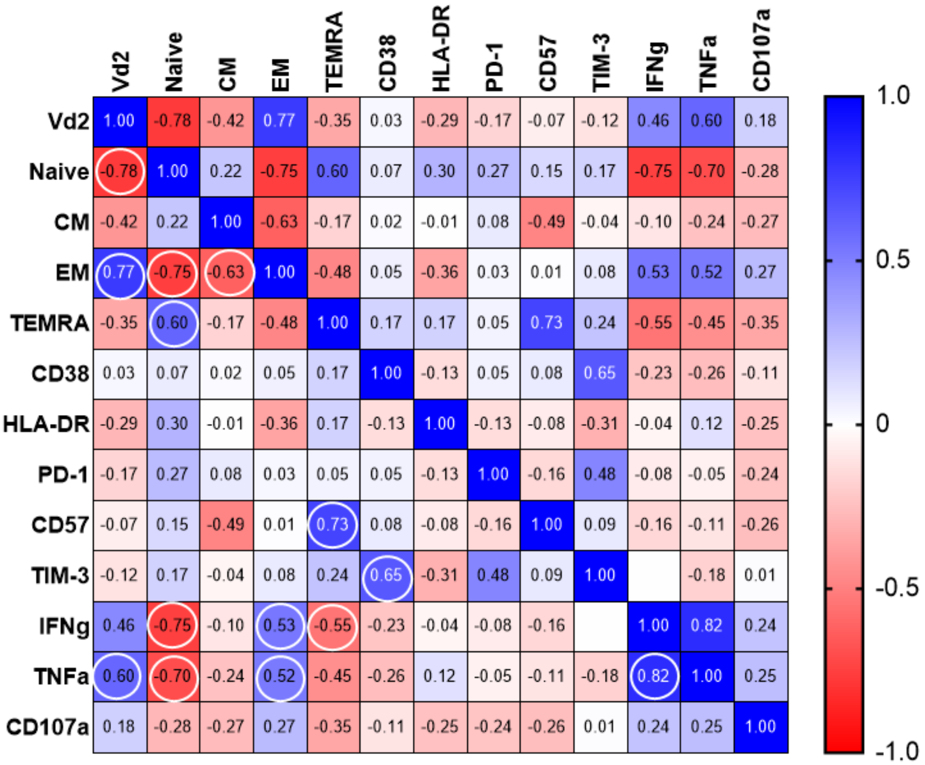

Supplementary Figure 5. **IFNγ-Vδ2-producing T-cells correlation with PD-1 exhaustion marker.** The correlation of exhaustion marker (PD-1) with IFNγ- producing-Vδ2 T-cells was reported in Mpox subjects at the early phase of infection (T1-T2) and at T3M (Spearman correlation, R:-0.58 and P=0.001).

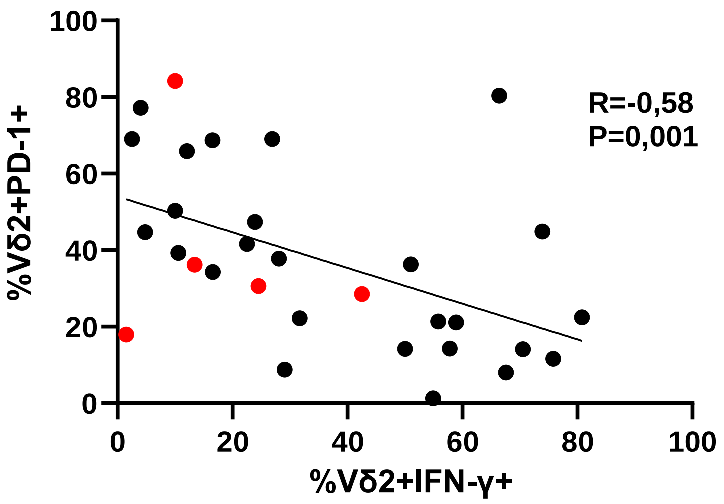

Supplement: Cimini E Supplements.pdf [file TEMI_A_2455585_SM8367.pdf]
